# Cardiovascular Protective Effects of Oral Hypoxia Inducible Factor Prolyl Hydroxylase Inhibitor Roxadustat in the Treatment of Type 4 Cardiorenal-Anemia Syndrome: Protocol of a Randomized Controlled Trial

**DOI:** 10.3389/fmed.2022.783387

**Published:** 2022-04-04

**Authors:** Yumin Wen, Yang Xu, Hui Tian, Sizhu Jiang, Guofang Jiang, Puqing Li

**Affiliations:** Department of Nephrology, Beijing Hepingli Hospital, Beijing, China

**Keywords:** cardiorenal-anemia syndrome, chronic kidney disease, anemia, heart failure, roxadustat

## Abstract

**Background:**

Patients with chronic kidney disease (CKD) are at high risk of developing heart failure and anemia, which is defined as type 4 cardiorenal-anemia syndrome (CRAS). CRAS aggravates the deterioration of both kidney and heart function, ultimately resulting in a high mortality. This study aims to examine the efficacy and safety of roxadustat in the treatment of type 4 CRAS.

**Methods and Design:**

This study is designed as a randomized, open-label, controlled trial. A total of 68 patients diagnosed with type 4 CRAS will be randomly divided into roxadustat group and erythropoietin with a 1:1 ratio. Participants in the roxadustat group will receive roxadustat with an initial dose of 70 or 100 mg three times a week, and participants in the erythropoietin group will receive subcutaneous injection of erythropoietin for 24 weeks, to maintain a hemoglobin ranging from 100 to 120 g per liter. The primary outcome is the change in heart function, including brain natriuretic peptide (BNP), 6-min walk test (6-WT), and left ventricular ejection fraction (LVEF). Secondary outcomes to be assessed include death, cardiovascular events, hospitalization regarding heart failure, Minnesota Heart Failure Quality of life scale (MLHFQ) score, New York Heart Association (NYHA) cardiac function grade, echocardiographic parameters including left ventricular diastolic diameter and volume (LVDD and LVDV) and ventricular mass (LVM), anemia related parameters, inflammatory parameters, and safety assessments.

**Conclusion:**

The findings of this study will provide potential evidence for roxadustat in CRAS management.

**Trial Registration:**

Chinese Clinical Trial Registry, ID: ChiCTR2100050031. Registered on 16 August 2021.

## Introduction

Type 4 cardiorenal-anemia syndrome (CRAS) is defined as a pathophysiological disorder whereby chronic kidney disease (CKD) leads to chronic cardiac dysfunction and anemia (1). The vicious circle between heart failure, anemia, and kidney disease contributes to a significantly worse prognosis and high mortality (2). Cardiovascular disease (CVD) is the leading cause of death in patients with estimated glomerular filtration rate (eGFR) <60 ml/min per 1.73m^2^ (3), while anemia is an independent risk factor for CVD in CKD patients. Compared with hemoglobin between 120 and 129 g/L group, the risk of heart failure in CKD patients with hemoglobin <100g/L increases by 1.99 times (4). For every 10g/L reduction of hemoglobin in dialysis patients, the risk of left ventricular dilatation increased by 42%, and the risk of new/recurrent heart failure increased by 18% (5). Despite the high prevalence and significance of renal anemia (RA), its management strategies are far from satisfactory. In China, only 39.8% of RA patients received erythropoiesis-stimulating agents (ESAs) treatment, 22.7% of patients initiated anemia-correction when hemoglobin decreased to less than 70 g/L, and only 8.2% of patients achieved a target hemoglobin of 110–120 g/L (6). Because of this, anemia-correction seems to be a reasonable approach to treat type 4 CRAS. ESAs together with iron supplements are conventional treatments for RA. Unfortunately, it was confirmed by the reduction of events by darbepoetin alfa in heart failure (RED-HF) study (7) that treatment with ESAs did not improve clinical outcomes in patients with systolic heart failure and mild-to-moderate anemia. On the contrary, the use of ESAs equivalent to more than 10,000 U/week of recombinant human erythropoietin (rHuEPO)/epoetin alfa will increase the risk of hypertension, stroke, thrombosis, thrombolysis in hemodialysis vascular access, and all-cause mortality (8). Iron overload due to excessive iron supplements also resulted in an increased risk of cardiovascular morbidity and mortality among hemodialysis patients (9). There is an urgent need to develop a safe and effective treatment for CRAS.

Roxadustat is a hypoxia-inducible factor-propyl hydroxylase inhibitor (HIF-PHI), developed as an agent for the treatment of RA (10). It can promote the production of endogenous EPO, increase the sensitivity of EPO receptor, diminish the level of hepcidin, and improve the iron homeostasis in CKD patients (11). Apart from its effectiveness in RA management, roxadustat displayed a lower risk of a composite cardiovascular endpoint including myocardial infarction, stroke, all-cause mortality, and unstable angina or congestive heart failure requiring hospitalization compared with epoetin alfa (12). Of note, this data is generally based on *post-hoc* analysis. Because of this, we designed a randomized controlled trial to assess the cardiovascular protective effects and safety of roxadustat in the treatment of type 4 CRAS.

## Methods and Analysis

### Study Design

**Figure 1:**
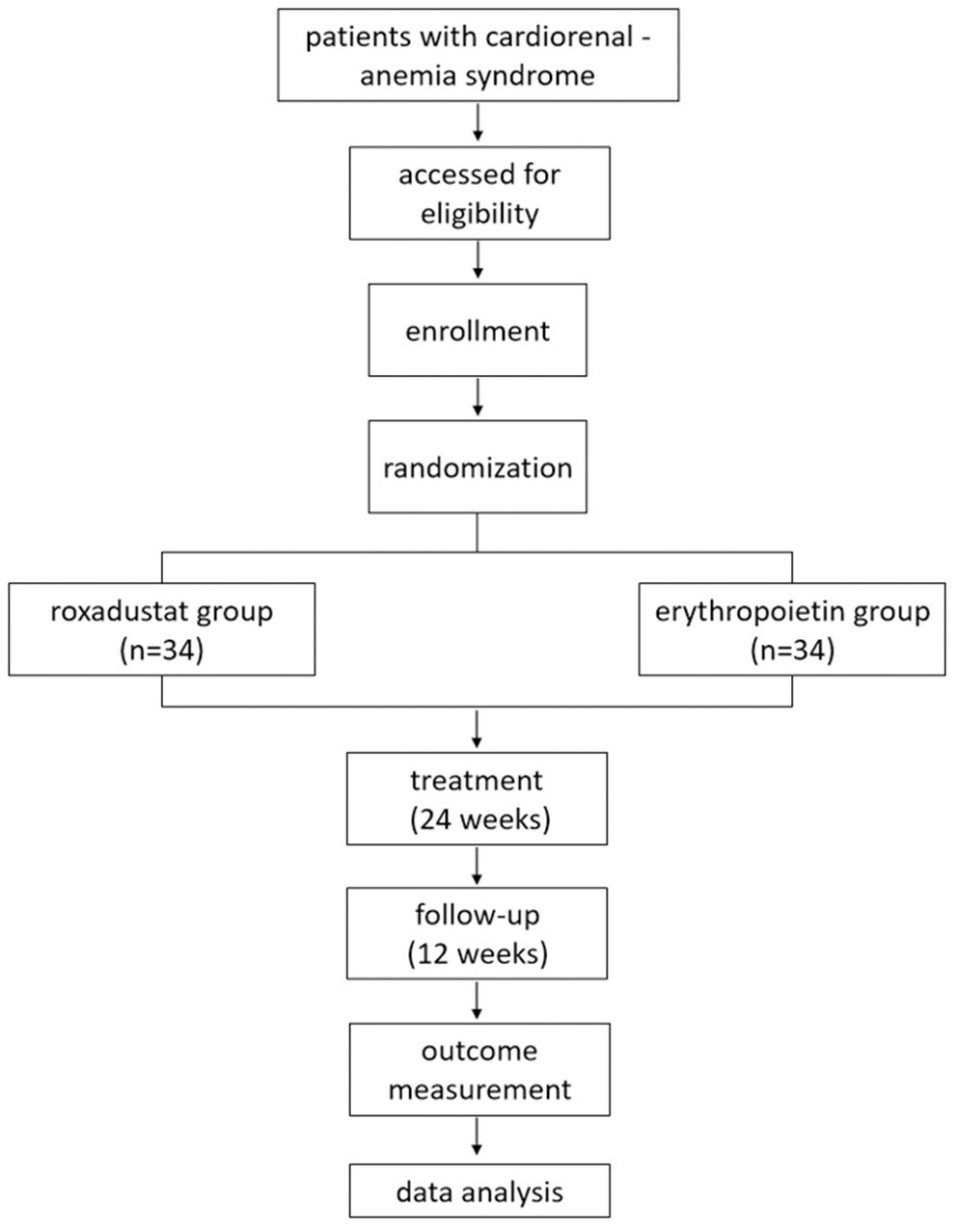
The flow chart of study design.

This trial is an open-label randomized controlled study designed to address the efficacy and safety of roxadustat in patients with type 4 CRAS. The study is comprised of a 24 week treatment period and a 12 week follow-up period, with visits at 0, 4, 12, 24, 28, and 36 weeks. The trial flow is illustrated in Figure 1. The outcome measurements and time points of data collection are demonstrated in Table 1.

**Table 1:** The schedule of enrollment, interventions, and assessments.

		**Study period**					
	**Enrollment**	**Allocation**	**Treatment**			**Follow-up**	
Time points (weeks)	−2	0	4	12	24	28	36
informed consent	√						
Eligibility screen	√						
Demographic data	√						
Medical history	√						
BNP		√	√	√	√		√
6-WT		√		√	√		√
LVEF		√		√	√		√
Death		√	√	√	√	√	√
CEs		√	√	√	√	√	√
MLHFQ scale		√		√	√		√
NYHA class		√	√	√	√	√	√
Echocardiography		√		√	√		√
Anemia parameters		√		√	√		
Inflammatory parameters		√		√	√		
Blood routine		√	√	√	√		
Urine routine		√	√	√	√		
Liver function		√	√	√	√		
electrocardiogram		√	√	√	√		
Adverse events		√	√	√	√	√	√

*BNP, brain natriuretic peptide; 6-WT, 6-min walk test; LEVF, left ventricular ejection fraction; CEs, cardiovascular events; MLHFQ, Minnesota Living with Heart Failure Questionnaire; NYHA, New York heart association. √, information collection, or parameter detection*.

### Participant Recruitment

Patients with CRAS as defined in the inclusion/exclusion criteria below will be invited to participate in the trial. Participants will be recruited from either the outpatient or inpatient of Beijing Hepingli hospital, Beijing, China. The recruitment was initiated in August 2021.

### Diagnostic Criteria

CKD and RA are defined by the Kidney Disease: Improving Global Outcomes (KDIGO) guideline (13), and chronic heart failure (CHF) is defined by the European Society of Cardiology (ESC) guideline (14). Type 4 CRAS is diagnosed in accordance with the Acute Dialysis Quality Initiative (ADQI) Consensus Conference (15).

### Inclusion Criteria

1. Age ≥18 years.
2. Meets the diagnostic criteria of type 4 CRAS.
3. New York Heart Association (NYHA) cardiac function grade II–IV, lasting for more than 4 weeks.
4. Non-dialysis patients with CKD stage 3a-5.
5. Hemoglobin level ≥ 70 and <100 g/L.
6. Voluntarily signed the informed consent form.

### Exclusion Criteria

1. Severe cardiovascular and cerebrovascular events in the last 3 months including myocardial infarction, acute coronary syndrome, stroke, epilepsy, or thromboembolism (e.g., deep vein thrombosis or pulmonary embolism).
2. Chronic inflammatory diseases that could impact erythropoiesis, such as systemic lupus erythematosus, rheumatoid arthritis, and abdominal diseases.
3. Anemia due to ailments other than CKD, such as myelodysplastic syndrome, multiple myeloma, hereditary hematopathy, etc.
4. Pregnant or lactating women.
5. Severe liver insufficiency.
6. Other serious systemic diseases.
7. Allergic to components of agents used in the study.
8. Mental disorder.

### Randomization, Blinding, and Allocation Concealment

The participants will be randomly allocated to the roxadustat group and erythropoietin group in a 1:1 ratio according to a computer-generated randomization sequence by a statistician who will be not involved in recruitment or data collection. This is an open-label study with investigators and participants unmasked to the information about allocation, while the statistician will be unaware of the treatment allocation. Any form of population-level summaries of outcome data will be prohibited before the end of the trial.

### Intervention

Eligible participants will be randomly assigned in a 1:1 ratio to receive either roxadustat or erythropoietin for 24 weeks.

1. All participants will receive optimized treatments for heart failure and renal anemia based on the 2019 ESC (16) and 2012 KDIGO practice guidelines. The detailed treatments are listed as follows:

(1) Treatment of heart failure: All participants will be recommended to take angiotensin-converting enzyme inhibitors (ACEIs)/angiotensin receptor blockers (ARBs), beta blockers, aldosterone blockers, or angiotensin receptor-neprilysin inhibitor (ARNi) as basic treatment for HF. The individualized strategy regarding the types and dosages of drugs will be obtained by their physicians. In order to reduce the adverse events of hyperkalemia and deterioration of renal function, ACEIs/ARBs administration should be avoided in patients with CKD stage 5 (eGFR <15 mL/min/1.73 m^2^), and ARNi administration should be avoided in patients with CKD stage 4 or 5 (eGFR <30 mL/min/1.73 m^2^), especially for patients with a serum potassium > 5 mmol/L. When the patients' serum potassium levels reach as high as 5.5 mmol/L, ACEIs/ARBs or ARNi should be halted.
(2) Iron deficiency treatment: All participants will receive 60 mg of oral elemental iron daily, with dose titration every 4 weeks to achieve a target transferrin saturation (TSAT) between 20 and 40%, as well as serum ferritin (SF) between 100 and 300 μg/L. The dose should be decreased if TSA > 50% or/and SF > 300 μg/L. If oral iron therapy is ineffective or intolerable after a 4-week treatment period, participants will be prescribed intravenous sucrose iron 100 mg/time, three times a week, and TSAT and SF will be evaluated again after a cumulative dosage of 1,000 mg.

1. Erythropoietin group

The erythropoietin (EPO) group will be treated with rHuEPO at an initial dose of 100–150 U/kg/week, divided into 2–3 subcutaneous injections, or 10,000 IU subcutaneously once weekly, with no restriction on the manufacturer of rHuEPO. The goal of initial EPO treatment is to increase hemoglobin by 10–20 g/L per month. The dose of rHuEPO can be increased by 60 U/kg/week, to maintain a target hemoglobin ranging from 100 to 120 g/L.

1. Roxadustat group

Participants randomized to roxadustat group will receive oral roxadustat (FibroGen, Co. Ltd, H20180024), with an initial dose of either 70 mg (body weight between 40 and 60 kg) or 100 mg (body weight ≥ 60 kg), 3 times a week. Based on the results of phase III clinical study of roxadustat in non-dialysis Chinese patients (17), the dose will be adjusted every 4 weeks following the rule displayed in Table 2, to maintain a hemoglobin level between 100 and 120 g/L.

**Table 2:** Dose adjustments of roxadustat.

**Δhemoglobin over the past 4 weeks (g/L)**	**Hemoglobin level (g/L)**			
	** <100**	**100– <110**	**110– <120**	**≥120**
< –10	Increase	Increase	No change	Hold, then resume dosing when hemoglobin <110 g/L at a dose that is reduced by 1 dose steps
–10 to 10	Increase	No change	Decrease	
>10	Increase	Decrease	Decrease	

*Investigator will adjust the dose in dose steps of 50, 70, 100, 120, 150, and 400 mg. The dose can be changed 1 step at a time, and the maximum dose is 2.5 mg/kg. Δhemoglobin, change in hemoglobin*.

### Outcome Measurement

1. Primary outcomes

The trial is designed to evaluate the improvement of heart function by roxadustat. Therefore, mean change in the brain natriuretic peptide (BNP), 6-min walk test (6-WT), and left ventricular ejection fraction (LVEF) estimated by echocardiography from baseline to 24 weeks will be applied as primary outcomes.

1. Secondary outcomes

(1) All-cause death and cardiovascular death.
(2) Cardiovascular events including non-fatal acute myocardial infarction and non-fatal stroke.
(3) Hospitalization regarding heart failure.
(4) Change in Minnesota Living with Heart Failure Questionnaire (MLHFQ) score from baseline to 24 weeks.
(5) Progress of cardiac function assessed by NYHA classification from baseline to 24 weeks.
(6) Echocardiographic parameters including left ventricular diastolic diameter and volume (LVDD and LVDV) and ventricular mass (LVM).
(7) Change in anemia-related parameters including the hemoglobin level, hemoglobin response rate (proportion of patients with hemoglobin elevation ≥ 10 g/L), serum iron, SF, TSAT, and hepcidin from baseline to 24 weeks.
(8) Change in inflammatory parameters including high-sensitivity C-reactive protein (hs-CRP), procalcitonin (PCT), interleukin-6 (IL-6), tumor necrosis factor a (TNF-a), and lipoprotein a [Lp (a)] from baseline to 24 weeks.
(9) Safety assessment consists of blood pressure, blood routine, urine routine, alanine aminotransferase (ALT), aspartate aminotransferase (AST), alkaline phosphatase (ALP), gamma-glutamyl transpeptidase (GGT), serum total bilirubin (TBIL), serum potassium, serum sodium, serum chlorine, and electrocardiogram.

### Adverse Events

Adverse events (AEs), comprising the precise time of occurrence, severity, duration, processing, and prognosis, will be recorded in the case report form at every study visit, and their correlation with the study medications will be assessed. The severity of AEs will be classified into mild, moderate, and severe. Severe AEs will be reported to the principal investigator and the ethics committee.

### Sample Size Estimation

Sample size is calculated to identify the significant difference in BNP after the 24-week intervention between groups. According to the previous published study (18), ESAs treatment could decrease BNP to 335±138 pg/ml. Our pilot study figured out that BNP level could decline to 234 ± 87 pg/ml with roxadustat. We applied the following formula to calculate the sample size.


$$\begin{array}{l} n=\frac{\left(1+\frac{1}{\text{k}}\right)\sigma^{2}}{{\left(\mu_{1}-\mu_{2}\right)}^{2}}\times {\left(\mu_{\alpha }+\mu_{\beta }\right)}^{2} \end{array}$$

The variable k is the sample ratio of roxadustat group and erythropoietin group. The variables μ_1_ and μ_2_ are the means in the roxadustat group and erythropoietin group. The total variance σ^2^ can be estimated by the sample variance *s*^2^ with the formula $s^{2}=\frac{s_{1}^{2}+ks_{2}^{2}}{1+k}$. The variables *s*_1_and *s*_2_ are the standard deviations in the roxadustat group and erythropoietin group. With a power of 90% (β = 0.10) and a one-sided level of significance of 0.05 (α = 0.05), n will be 27. Assuming a dropout rate of 25%, the sample size for patients in each group is 34.

### Statistical Analysis

Statistical analysis will be performed using SPSS version 20.0 (IBM Corp., Armonk, NY) software. This trial will include three analysis sets: the full analysis set, the per-protocol analysis set, and the safety analysis set. The full analysis set will include participants who receive the randomized treatment for at least 4 weeks. The per protocol analysis set will be restricted to participants who complete the 24-week treatment. Participants who have made at least one visit after randomization will be involved in the per-protocol analysis set group. Missing data will be processed following the principle of the last observation carried forward (LOCF). Normally distributed continuous variables will be expressed as the mean ± standard deviation, while non-normally distributed continuous variables will be expressed as median and inter quartile range. Categorical variables will be presented as numbers and percentages. Either independent *t*-tests (or the Mann–Whitney *U*-test) for continuous variables or chi-square (or Fisher exact) for categorical data will be applied to compare the differences between the two groups. Analysis of differences between pre- and post-treatment will be performed by paired Student *t*-test. As for primary outcomes of BNP, 6-MT, and LVEF, repeated measures analysis of variance (ANOVA) will be applied to detect the effects of treatment and time course. For safety analysis, the chi-square test or Fisher exact test will be used to analyze the incidence of AEs and the change of laboratory parameters between the two groups. All tests are 2-sided, and *P* < 0.05 will be considered significant.

## Discussion

The concept of CRAS, formerly known as cardio renal syndrome, has gained considerable interest over the last decade (19). Despite worldwide concern, the mechanistic framework of CRAS remains to be illustrated, and there is a lack of effective treatment (20). Several pilot or small sample studies (21, 22) indicated that ESAs alone or combined with iron supplement could reduce BNP and cardiovascular hospitalization in CKD patients with anemia and heart failure. Unfortunately, these findings were overturned by converse results of well-designed and large sample clinical trials (23). In the 2021 ESC guideline for heart failure, ESAs were excluded from the treatment of heart failure related anemia (24). Although the underline mechanism has not been elucidated, it is suggested that exogenous erythropoietin generated by ESAs can largely increase C-terminal fibroblast growth factor 23 (FGF23) level (25), which is significantly associated with left ventricular hypertrophy and an increased risk of mortality. Roxadustat is an oral anemia-correction agent function that increases endogenous erythropoietin by inhibiting propyl hydroxylase induced hypoxia-inducible factor degradation (26). With the advantages of endogenous erythropoietin generation and FGF23 lowering (27), roxadustat is a reasonable candidate in CRAS treatment, but there is a lack of relevant clinical evidence in this regard. Moreover, roxadustat may have additional effects of cholesterol-lowering (10) and could protect experimental animal from myocardial ischemia-reperfusion injury (28). It is reasonable to assume that roxadustat is a promising agent for CRAS with multiple mechanisms involved. This study will be the first attempt to evaluate the potential benefit of roxadustat in patients with CRAS.

Former studies that focused on anemia-correction in CRAS mainly included patients with mild anemia, and the target hemoglobin level reached as high as 130 g/L. High hemoglobin levels might be partially responsible for adverse events such as stroke, hypertension, and thrombosis (29). In this study, we will recruit patients with a much lower hemoglobin level of 70–100 g/L, who will benefit more from anemia-correction. To decrease the incidence of adverse events resulting from excessive increase of hemoglobin, the target hemoglobin level in this study will be 100–120 g/L.

BNP and N-terminal pro-B-type natriuretic peptide (NT-proBNP) have been shown to be independent predictors of mortality (all-cause and cardiovascular) in advanced heart failure despite different cutpoints, time intervals, and prognostic models. In this study BNP is designed as one of the primary outcomes and is used as surrogate to calculate the study sample size, while NT-proBNP, with a relatively longer plasma half-life, is adopted as a surrogate in plenty of clinical trials. So, we think either BNP or NT-proBNP is acceptable as a prognostic marker of CHF.

As an open-label study, both the participants and investigators will be aware of the assignment and treatment. To ensure the quality of this study and minimize bias, the analysis of data will be performed by an independent statistician who is not involved in the study and blind to the allocation of treatment.

There are some limitations to this study. First, the study is single-centered and open-labeled, indicating there might be increased bias. Second, since most of the participants are unlikely to reach hard clinical endpoints, such as all-cause mortality or cardiovascular mortality, in the short study duration of 36 weeks, primary outcomes only include surrogate endpoints.

We expect that the results of this study will provide a scientific rationale for the use of roxadustat in improving the heart function of type 4 CRAS.

## Ethics Statement

The studies involving human participants were reviewed and approved by the Ethics Committee of Beijing Hepingli hospital. The patients/participants provided their written informed consent to participate in this study.

## Author Contributions

YW and YX: drew up the design of the study. HT and GJ: improvement of the research design. SJ and PL: manuscript draft and trial registration. All authors contributed to the article and approved the submitted version.

## Funding

This study was supported by the National Natural Science Foundation of China (81804055), and Dongcheng District health science and technology program of Beijing (DWJY[2021]-5).

## Conflict of Interest

The authors declare that the research was conducted in the absence of any commercial or financial relationships that could be construed as a potential conflict of interest.

## Publisher's Note

All claims expressed in this article are solely those of the authors and do not necessarily represent those of their affiliated organizations, or those of the publisher, the editors and the reviewers. Any product that may be evaluated in this article, or claim that may be made by its manufacturer, is not guaranteed or endorsed by the publisher.
